# Genome-Wide Characterization and Abiotic Stresses Expression Analysis of Annexin Family Genes in Poplar

**DOI:** 10.3390/ijms23010515

**Published:** 2022-01-03

**Authors:** Hui Wei, Ali Movahedi, Guoyuan Liu, Yixin Li, Shiwei Liu, Chunmei Yu, Yanhong Chen, Fei Zhong, Jian Zhang

**Affiliations:** 1 Key Laboratory of Landscape Plant Genetics and Breeding, School of Life Sciences, Nantong University, Nantong 226000, China; 15850682752@163.com (H.W.); cjqm1989@126.com (G.L.); liyixinlyx0@163.com (Y.L.); 2009110160@stmail.ntu.edu.cn (S.L.); ychmei@ntu.edu.cn (C.Y.); chenyh@ntu.edu.cn (Y.C.); fzhong@ntu.edu.cn (F.Z.); 2 Co-Innovation Center for Sustainable Forestry in Southern China, Key Laboratory of Forest Genetics & Biotechnology, Ministry of Education, College of Biology and the Environment, Nanjing Forestry University, Nanjing 210037, China; ali_movahedi@njfu.edu.cn; 3 College of Arts and Sciences, Arlington International University, Wilmington, DE 19804, USA

**Keywords:** poplar annexin, duplication, tissue-specific expression, abiotic stress

## Abstract

Poplar is an illustrious industrial woody plant with rapid growth, providing a range of materials, and having simple post-treatment. Various kinds of environmental stresses limit its output. Plant annexin (ANN) is a calcium-dependent phospholipid-binding protein involved in plant metabolism, growth and development, and cooperatively regulating drought resistance, salt tolerance, and various stress responses. However, the features of the PtANN gene family and different stress responses remain unknown in poplar. This study identified 12 *PtANN* genes in the *P. trichocarpa* whole-genome and PtANNs divided into three subfamilies based on the phylogenetic tree. The PtANNs clustered into the same clade shared similar gene structures and conserved motifs. The 12 *PtANN* genes were located in ten chromosomes, and segmental duplication events were illustrated as the main duplication method. Additionally, the PtANN4 homogenous with AtANN1 was detected localized in the cytoplasm and plasma membrane. In addition, expression levels of *PtANNs* were induced by multiple abiotic stresses, which indicated that PtANNs could widely participate in response to abiotic stress. These results revealed the molecular evolution of *PtANNs* and their profiles in response to abiotic stress.

## 1. Introduction

Plants may meet with adverse environments during their cycles of growth and development [1]. Drought and salinity are the main environmental factors that affect the geographical distribution of natural plants and limit agricultural and forestry output yield. Meanwhile, the increasing frequency of extreme weather results in the aggravation of the adverse effects of abiotic stress on plants [2]. Due to plants suffering from low water tolerance, more water may be used to irrigate, which increases the burden on the environment. Therefore, agricultural and forestry production needs to breed stress-tolerant rice cultivars. However, plants can recognize and sense adverse environments during long-term evolution based on various signal transduction pathways, such as the abscisic acid (ABA) signal transduction pathway [3,4]. Cytosolic ABA signaling cascade is composed of pyrabactin resistance 1 (PYR1)/PYR1-like (PYL), protein phosphatase 2C (PP2C), and sucrose non-fermenting1-related protein kinase2 (SnRK2). In the ABA-dependent signal transduction pathway, the SnRK2.6 phosphorylated by PP2C results in the inactivation of SnRK2.6 when the ABA is not present in the cytosol. However, integration of ABA and PYR/PYL can inhibit the PP2Cs, which leads to activating SnRK2.6 and initiating ion channels and expression of ABA-responsive genes [5,6]. In addition, the promotion of ABA contents results in stomatal closure coupled with transpiration and water loss reduction. Moreover, ABA accumulation leads to activation of the Ca^2+^ channel in the plasma membrane and improved free Ca^2+^ concentration in plant cytoplasm in response to environmental stresses [7,8].

Annexins (ANNs) localized on the cytosolic membrane are a multigene family of Ca^2+^-dependent proteins that maintain intracellular and extracellular Ca^2+^ homeostasis [9]. The distribution of ANNs occurs widely in some eukaryotes and prokaryotes with various numbers, and the first ANN family members were characterized in *Arabidopsis thaliana* [10]. Subsequently, ten *ANN* genes were identified from *Oryza sativa* and *Medicago sativa*, respectively [11]. Given ANN structure, these proteins consist of a core domain including four-fold ANN repeats (I, II, III, and IV) with approximately 70 amino acids and an N-terminal structure with different sequences and lengths [12]. For plant ANN repeats, I and IV include an inline protein sequence of GXGT-(38 variable amino acid residues)-D/E considered Ca^2+^ binding sites. Still, deficiency of Ca^2+^ binding sites on ANN repeats II and IV [13,14]. The ANN repeats have relatively higher similarities among ANN members from different species, indicating a specific evolutionary relationship. The divergences among amino acid sequence and composition of ANN members mainly lie in the N-terminal structures. Therefore, it is speculated that the different characterizations and functions among ANN members may result from the difference in N-terminal structures. A study illustrated that the N-terminal structure of *Capsicum annuum* ANN (ANNCa32) could interact with its core domain (ANNCa32 repeat I), indicating that the regulatory functions of plant ANN N-terminal structure are relatively conservative [15]. In addition, ANN repeats can form curved disks, and the convex surfaces of curved disks contain Ca^2+^ binding sites. The convex surface binding Ca^2+^ faces the cell membrane when the annexin binds to phospholipids. The concave surface is toward the cytoplasmic matrix, making ANN interact with other proteins and other molecules in the cytoplasm [9].

Moreover, plant ANNs have some sites of post-translational modifications, such as phosphorylation site (Ser, Thy, Lys), S-glutathionylation site (Glu), glycosylation site (N-Gly), and myristoylation site (N-Myr) [16,17]. A few studies showed that plants could respond to environmental changes based on the ANN post-translational modifications. The post-translational improvements of *A. thaliana* ANN1 (AtANN1) by phosphorylation can improve the peroxidase activity, but dephosphorylation reduces the peroxidase activity [12]. Additionally, the down-regulation of Ca^2+^-binding efficiency resulting from AtANN1 glutathionylation could affect membrane interaction [12]. The interaction relationship among ANN and protein kinases in *O. sativa* illustrated that some protein kinases (protein kinase C (PKC), mitogen-activated protein kinase (MAPK), and cyclic AMP-cyclic GMP (cAMP-cGMP) coordinate with phosphatase to regulate the post-translational modification of rice ANN and could be involved in the Ca^2+^-dependent MAPK signaling [18].

The various studies reported that ANNs have ATPase/GTPase and peroxidase activities coupled with regulation of Ca^2+^-binding activity and contribute to regulating a large number of essential physiological processes in response to environmental stresses [19,20,21,22]. For example, the relative expression of rice Nagina22 (N22) *ANN* was improved under the drought treatment [23]. *Triticum aestivum Anns*, *P39* and *P22.5* expression levels increased when exposed to low-temperature stress, illustrating that TaANNs might participate in low-temperature signal transduction [24]. *Arachis hypogaea* ANNs were speculated to be involved in drought, salt, heavy metals, and low-temperature stresses depending on the analysis of *AhANNs* expression accumulations under the above stresses [25]. In addition, AtANN1 interaction with AtANN4 took essential roles in response to salt and drought treatments under the long-day condition and influenced the photoperiod of Arabidopsis [26]. *OsANN3* expression level was induced under polyethylene glycol (PEG), and abscisic acid (ABA) treatments and overexpression of *OsANN3* in rice improved the survival rates of rice under drought stress, suggesting that OsANN3 conferred drought stress tolerance at the seed germination stages. Additionally, overexpression of *OsANN3* in rice increased the number and length of rice roots and stomatal closure, and reduced water loss by regulating the ABA-dependent stress response pathway [22]. Overexpression of *Nelumbo nucifera ANN* in Arabidopsis improved the heat tolerance and hydrogen peroxide activity, released lipid peroxidation, and reactive oxygen species (ROS) in transgenic Arabidopsis seeds were decreased [27]. AtANN8 could respond to salt and dehydration stresses and alleviate damage on those stresses in Arabidopsis [28]. Interaction between soybean GmANN and glutathione S-transferase (GmGST) responded to high temperature and high humidity (HTH) stress. It was concluded that it formed plant seed vitality [29]. GhDsPTP3a could interact with GhANN8b, which plays a positive role in the response of cotton to salt stress. Salt stress-induced phosphorylation of GhANN8b and GhANN3a, respectively, and GhANN8b and GhANN3a conversely regulated Ca^2+^ influx and Na^+^ efflux [30]. The increased cell membrane damage caused by cold stress was associated with the inhibition of transcript levels of *ZmANN33* and *ZmANN35*, and the rescue of cell membrane accompanied the rapid recovery of *ZmANN33* and *ZmANN35* expression levels. The Arabidopsis seedlings overexpressed *ZmANN33* and *ZmANN35* had better growth trends than wild-type (WT) Arabidopsis seedlings under the low temperature, illustrating that ZmANN33 and ZmANN35 play a positive role in response to low temperature [31]. Drought, heavy metal stress, and salt stress can lead to peroxide accumulation in plant cells and result in plant cell damages or large numbers of tissue and cell death. ANN plays an essential role in maintaining the stability of membrane structure and cell secretion and has been proved to have peroxidase activity. In addition, Ca^2+^ is closely related to the transcriptional regulation of the *ANN* gene, especially in the abiotic stress response, indicating that ANN is closely involved in environmental stress. Taken together, plant ANNs are involved in various kinds of physiological processes and play essential roles in response to environmental stresses.

Poplars have essential values as vital elements in industrial products and environmental ecology. They have unique profiles and characterizations, such as primary and secondary growth and metabolism and response to environmental stresses. Poplars show some resistance systems, including cell structure changes and signal transduction [32,33,34]. They are generally exposed to recurrent damages by various biotic and abiotic stresses. Therefore, the development and discovery of stress response theories to avoid or reduce injuries imposed by environmental stress are essential for poplars. Although the ANN gene family has been considered a vital stress regulator in model species, its molecular characterization and evolution remain less clear in woody plants. Here, the molecular characterization and function of the poplar ANN gene family was systematically analyzed. The 12 putative *PtANN* genes from *Populus trichocarpa* were cloned, and molecular evolution, protein structures, genes composition, and *cis*-elements were analyzed. Additionally, tandem and segmental duplication among poplar *ANN* genes and collinearity among *ANN* genes from different species were determined based on a comprehensive genome synteny analysis. The subcellular localization analysis was performed to determine the localization of PtANN. Furthermore, the expression patterns of *PtANN* members across different tissues and under divergent treatments were analyzed. In summary, the present study provides insight into the characterizations and structures of PtANNs and lays the foundation for illustrating the biological function in response to abiotic stress.

## 2. Results

### 2.1. Identification of Putative ANN Family Members in Poplar

Genes, proteins, and coding DNA sequences (CDS) annotation in *P. trichocarpa* were downloaded from the Phytozome database. HMMER software version 3.0 [35] was applied to identify the poplar ANN members based on the Pfam database (PF00191). Additionally, the Arabidopsis ANN sequences were obtained from Clark et al. [10] and Cantero et al. [36] (Supplementary Table S1), as a query to search the PtANN members in *P. trichocarpa* genome. Then, the SMART and NCBI CDD were used to ensure that whether each putative PtANN contains a complete ANN domain or not. In summary, 12 *PtANN* genes were identified from the poplar genome based on Pfam and sequence similarity with AtANN proteins. In addition, according to chromosome localization of *PtANNs*, 12 *PtANN* genes were named *PtANN1*-*PtANN12* (Supplementary Table S1).

Based on the characterization of 12 PtANNs, the length of 12 PtANN proteins ranged from 312 aa to 329 aa, indicating that little diversity within the length of PtANN members. We speculated that the little divergence in length results from the 4 ANN repeats in C-terminal and variable sequences in the N-terminal structure. The predicted isoelectric points (pI) ranged between 5.69 (PtANN1) and 9.23 (PtANN10), suggesting PtANN proteins not only belong to acidic proteins but also reside the alkaline proteins. The instability index varied from 31.39 to 52.73. PtANN2, PtANN8, PtANN9, and PtANN11 were thought to be unstable proteins, while others were considered stable proteins. The aliphatic index of PtANNs ranged from 84.26 (PtANN11) to 97.6 (PtANN5) had diversity features. Additionally, the GRAVY values of PtANNs were predicted to be negative, representing that all PtANNs possess hydrophilic characteristics. Subcellular localization of PtANNs was speculated to be localized in the cytoplasm (Supplementary Table S2). 

### 2.2. ANN Sequences Alignment and Phylogenetic Evolution Analysis of ANN Members

Through the multiply alignment of poplar, Arabidopsis, and rice ANNs, the poplar ANN family proteins had relatively high identification with Arabidopsis and rice ANNs, suggesting that the ANN family is relatively conservative in the process of evolution. Additionally, poplar ANNs contained four relatively conserved repeat domains (Supplementary Figure S1), and each repeat contained approximately 70 amino acid residues. Both the I and IV repeat domains had the dominant Ca^2+^ binding site (G/KXGT-38-D/E), the main structural features of plant ANNs, while no Ca^2+^ binding site was found in II and III repeat domains. In addition to the Ca^2+^ binding site, poplar ANNs also contained some critical conserved sites related to function. For example, IRI site binding to F-actin was displayed in repeat domain III; DXXG site binding to GTP was found in repeat domain IV; S3 clusters (MCCY) were associated with ANN peroxidase activity; histidine40 (His40) residues binding to hemoglobin were involved in a redox reaction, and tryptophan (Trp) residues participated in promoting ANN binding to membrane independent Ca^2+^ (Supplementary Figure S1). The above observations showed that poplar ANNs are relatively conserved with other plants ANNs in structure.

**Figure 1:**
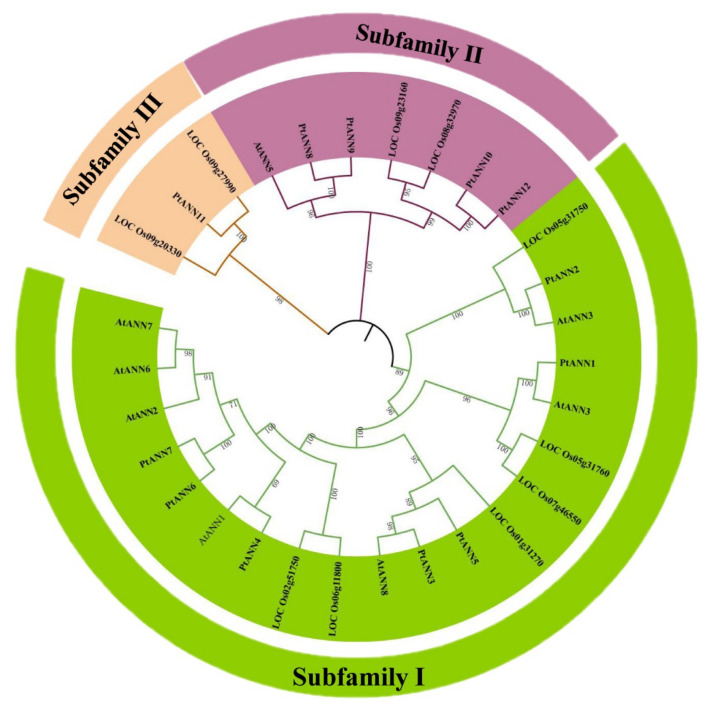
Phylogenetic tree analysis of ANN proteins among poplar, Arabidopsis, and rice. The MEGA7 software was applied to draw the neighbor-joining (NJ) method tree with 1000 bootstrap replicates. Lines with Roman numerals represent different ANN subfamilies. The Arabidopsis, rice, and poplar ANN proteins can be clustered into three clades.

To discover the molecular evolution of PtANNs, a phylogenetic tree with neighbor-joining (NJ) method was constructed by PtANN protein sequences (Supplementary Figure S2). To further clarify the evolutionary relationship and putative functions of PtANNs, the NJ method was also performed to establish a phylogenetic tree with 12 PtANNs, 8 AtANNs, and 10 OsANNs (Figure 1). According to the phylogenetic tree, the PtANN gene family was divided into three major subfamilies: I, II, and III. Among them, subfamily I consisted of PtANN1–7; subfamily II composed of PtANN8–10 and PtANN12; subfamily III contained PtANN11 (Supplementary Figure S2). Additionally, the phylogenetic tree displayed the evolution relationship among poplar, Arabidopsis, and rice fell into three distinct subfamilies, illustrating that ANN may originate from some ancestral gene and is evolved into three subfamilies in the process of evolution (Figure 1). In addition, the clustering relationship of poplar, Arabidopsis, and rice ANNs was relatively complex. ANNs of poplar, Arabidopsis, and rice were widely distributed at the phylogenetic tree, and PtANNs were interspersed and distributed in each clade, showing the complexity of the evolutionary relationship of ANNs. In most cases, the PtANN members were first grouped with AtANN proteins and then grouped with OsANN proteins within each clade, which indicated that ANN from *P. trichocarpa* might have a closer evolutionary relationship with Arabidopsis ANNs. However, a small part of PtANNs was clustered into OsANNs in clades, indicating that the ANN evolutionary relationship was somewhat divergent from the relationship among monocots and dicots.

### 2.3. Analysis of PtANN Phosphorylation Site and 3D Structure

The NetPhosK 3.0 Server was used to analyze the post-translational modification of the kinase phosphorylation site, and the result showed that PtANN family proteins contain different types of phosphorylation sites (Supplementary Table S3). The serine phosphorylation sites had made up a considerable part of PtANN phosphorylation sites, followed by threonine phosphorylation and tyrosine phosphorylation. Moreover, the phosphorylation probabilities of serine, threonine, and tyrosine in PtANN were 50.99%, 38.61%, and 10.4%, respectively. In the PtANN family proteins, the phosphorylation mode of PtANN6, PtANN7, and PtANN12 was the most likely protein kinase A (PKA) phosphorylation. At the same time, other PtANNs may apply the protein kinase C (PKC) phosphorylation mode. There were ten types of potential kinase phosphorylation mode in PtANN1, namely PKC, p38MAPK, cyclin-dependent kinase 5 (Cdk5), glycogen synthase kinase 3 (GSK3), DNA-dependent protein kinase (DNA-PK), ribosomal S6 kinase (RSK), PKA, cell division cycle2 (cdc2), casein kinase II (CKII), and CKI phosphorylation. PKC phosphorylation occurred ten times in PtANN1 phosphorylation mode, and the highest score (0.873) appeared on Ser44. Additionally, there were nine types of putative kinase phosphorylation mode in PtANN2, namely CKI, CKII, cdc2, PKC, PKA, PKG, RSK, p38MAPK, and EGFR phosphorylation. The CKII and PKC phosphorylation appeared the highest frequency of phosphorylation in PtANN2 phosphorylation, and Thr195 site had the highest score (0.866). In addition, PtANN9 has 11 types of kinase phosphorylation, namely PKC, DNAPK, ATM, PKA, CKII, INSR, p38MAPK, cdc2, CKI, EGFR, and RSK phosphorylation. PKC phosphorylation in PtANN9 reaching 14 times had the highest number of phosphorylation, and the highest score (0.819) was PKC on Thr91.

The three-dimensional (3D) structure prediction of PtANN proteins showed that PtANN proteins are composed of coils and helixes (Supplementary Figure S3). The helixes occupy a significant part of ANN structures, while coils only account for a small amount of ANN structures. The PtANNs were divided into three subfamilies based on the phylogenetic tree. PtANNs belonging to subfamily I shared a similar 3D structure, and the same result was found in subfamily II and III. Based on the comparative analysis of 3D structure and evolution of PtANN members, the PtANNs clustered in three different clades of the evolutionary tree showed the divergent 3D structure, suggesting that putative various features and functions might exist in members distributed in other subfamilies. In addition, the IRI site, S3 clusters, His residues, and Trp residues displayed a somewhat variance among PtANN members. For example, both PtANN4 and AtANN1 had the IRI site, S3 sites, His residues, and Trp residues, which suggested the features and functions of PtANN4 might be similar with AtANN1. However, the F-actin binding domain (IRI) was not identified in PtANN1, and PtANN2 had the S3, IRI, and Trp site except for the DXXG domain. All these observations indicated that there are differences in some special functional sites of PtANNs, in addition to the common conserved domain. Those functional site divergences might result in various functions of PtANN members in the process of evolution.

### 2.4. Analysis of *PtANN* Gene Structures and Conserved Motifs

**Figure 2:**
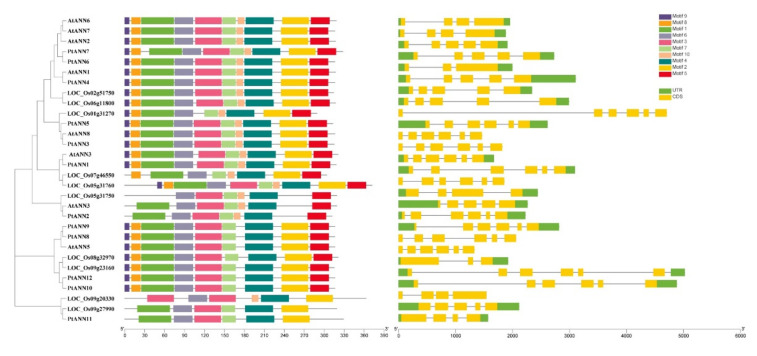
The PtANN conserved motifs and *PtANN* gene structures analysis. The motif compositions and distributions of PtANNs were identified by MEME, and different colorful rectangles indicated divergent motifs. The *PtANN* gene structures were performed to identify using genome database and annotation, and the solid black lines, green and yellow rectangles implied intron, 5′-/3′-UTR, and exon, respectively.

To illustrate the features of *PtANNs*, the intron/exon patterns of poplar, Arabidopsis, and rice *PtANNs* were investigated based on the poplar, Arabidopsis, and rice genomes and innovations (Figure 2). The genomic *PtANN* sequences illustrated the number of exons ranging from four to six, and the number of introns changed from three to five. Most of them usually had six exons and five introns. The length of *PtANN12* introns was longer than other *PtANN* introns. Moreover, *PtANNs* belonging to subfamily I shared the relatively similar intron/exon patterns, while the divergent intron/exon patterns were identified in different subfamilies. In addition, the MEME online tool was used to predict conserved motifs of PtANN proteins (Figure 2). The number of PtANN motifs was distinctive, ranging from 1 to 10, and most of PtANNs shared five to eight motifs. Generally speaking, the PtANNs in the same evolutionary clade have certain similarities in the kinds and relative positions of motifs. For example, PtANN8–10 and PtANN12 clustered in the same evolutionary clade shared similar motif compositions. In contrast, the types of motifs showed divergences in the different evolutionary clade, such as PtANN2 and PtANN5. Moreover, the result of PtANN in the same evolutionary clade possessing the similar motif compositions was consistent with PtANNs family phylogenetic clustering, which further supported the evolutionary relationship among each PtANN member.

### 2.5. Analysis of *PtANN Cis*-Acting Elements and Putative Interaction of Protein-Protein

**Figure 3:**
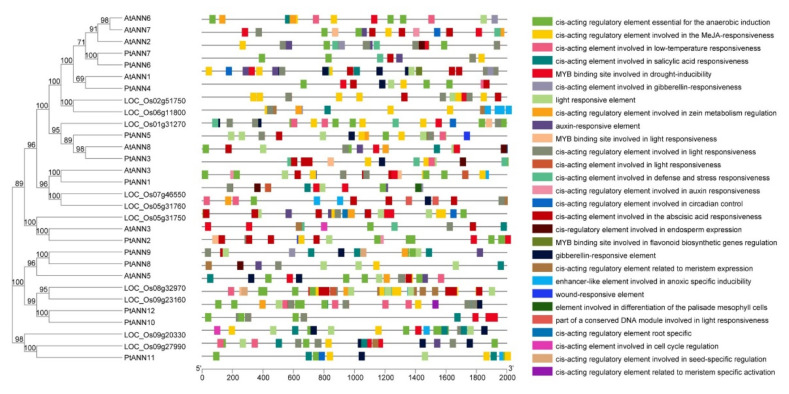
The predicted *cis*-elements in *PtANN* gene promoters. PlantCARE analyzed promoter sequences (−2000 bp) of poplar, Arabidopsis, and rice *ANN* genes. The different colored rectangles indicated divergent *cis*-elements, and the solid black lines implied *PtANN* promoters.

The *cis*-acting promoter elements are short DNA sequences presented on the promoters. They ensure the specificity of the gene expression response because different transcription factors specifically recognize them. The PlantCARE was applied to illustrate the *cis*-acting promoter elements of *PtANNs*, and various kinds of *cis*-acting elements were identified in *PtANN* promoters (Figure 3). The *cis*-acting elements are involved in plant development, response to abiotic stress and hormone, and putative *cis*-acting elements including light-, gibberellin- (GA), salicylic acid- (SA), auxin-, anaerobic, abscisic acid- (ABA), methyl jasmonate- (MeJA) and low-temperature responsiveness. Most *PtANN* promoter elements were identified as involved in hormone and light response. The *cis*-acting elements related to endosperm and tissue development were also found in *PtANN* promoters. The *cis*-acting elements associated with SA-responsiveness were located in the promoters of *PtANN1*, *PtANN2*, *PtANN7*, *PtANN8*, *PtANN10*, and *PtANN11*. The *cis*-acting elements that respond to GA were found in the *PtANN1–5*, *PtANN7*, *PtANN9*, and *PtANN11* promoters. The *cis*-acting elements involved in ABA-responsiveness were illustrated in the *PtANN2–7* promoters. The *cis*-acting elements connected with MeJA-responsiveness mainly existed in the promoters of *PtANN3–5*, *PtANN6*, *PtANN8*, and *PtANN11*. The *cis*-acting elements that might be associated with low-temperature responsiveness were found in the *PtANN3*, *PtANN5*, *PtANN7*, and *PtANN10* promoters. All the observations of *PtANN cis*-acting elements indicated that *PtANN* genes might be involved in environmental stresses and hormone regulation and play an essential role in physiological and developmental processes.

### 2.6. Chromosomal Localization and Collinearity Analysis of PtANNs

According to the poplar genome annotation, the 12 *PtANN* genes were identified to localize in ten chromosomes (Supplementary Figure S4). The distribution of *PtANN* genes mapped on each chromosome was relatively independent and irregular. *PtANN1–3* was present on chromosome 1, while *PtANN* genes were absent on chromosomes 4, 6, 9, 11, and 14, and other chromosomes only contained one *PtANN* gene, respectively.

**Figure 4:**
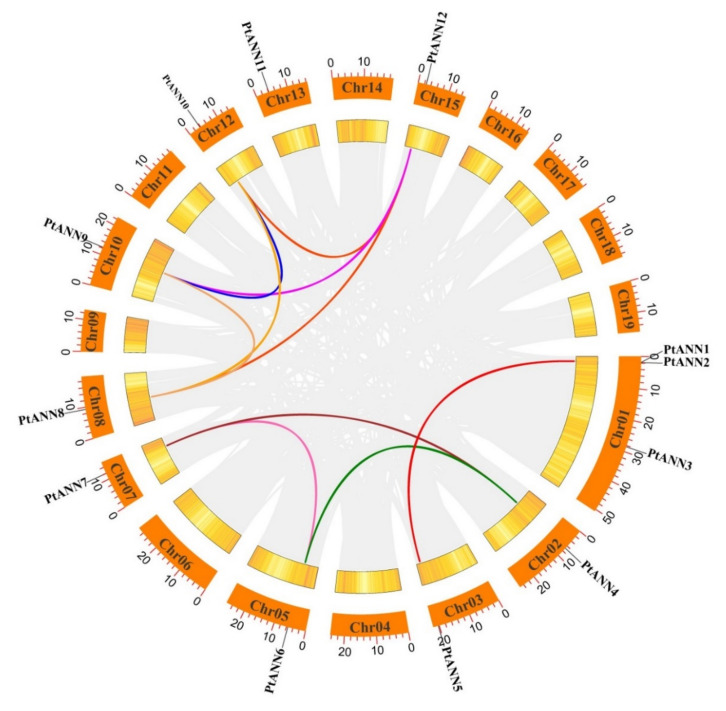
Synteny analysis of *PtANN* genes. The orange rectangles indicated chromosomes 01–19. The yellow gradient lines in rectangles implied gene densities on chromosomes. Gray lines in the circle showed segmental duplication events in poplar, and colored lines indicated collinearity events of *PtANN* members.

To further understand the evolutionary relationship of *PtANN* genes and the evolutionary origin of *ANN* genes, the microsynteny analysis within the poplar genome and among different species was performed using MCScanX and TBtools. The duplication events containing tandem duplication, segmental duplication, and whole-genome duplication played an essential role in plant evolution. Among 12 *PtANN* genes, no pairs of *PtANN* genes were identified as tandem duplication, which signified tandem duplication was not involved in *PtANN* expansion. In addition, segmental duplication surveys revealed ten gene pairs of *PtANN* genes mapping on chromosomes 1–3, 5, 7, 8, 10, 12, and 15 (Figure 4). The nonsynonymous (Ka) and synonymous (Ks) ratios were used to determine the selection pressure of gene duplications. The value of Ka/Ks on the *PtANN* genes usually is lower than 1, implying that *PtANNs* experience strong purifying selection during the process of evolution.

**Figure 5:**
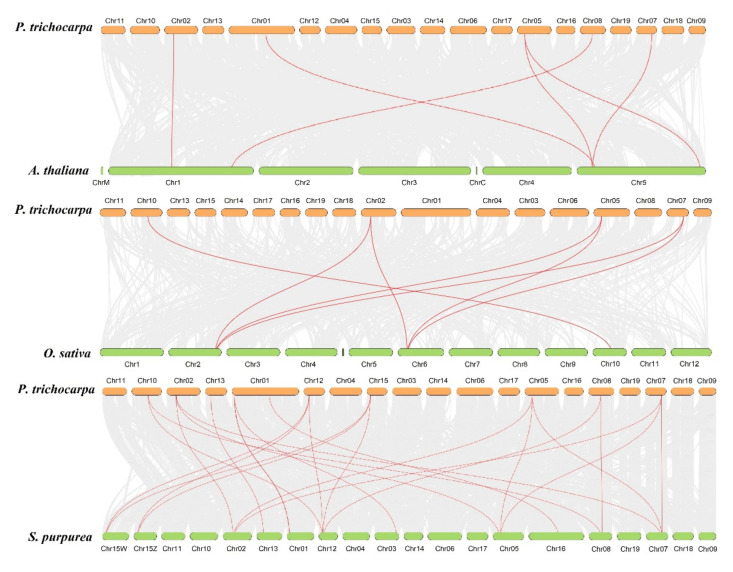
Extra-genomic collinearity related to PtANN gene family in *P. trichocarpa*, *A. thaliana*, *O. sativa*, and *S. purpurea*. Gray lines in extra-genome were presented at collinear blocks, and the colored lines implied synteny blocks of *ANN* genes.

To further explore the evolutionary origin among *PtANN*, *AtANN*, *OsANN*, and *SpANN* members, a syntenic map of *P. trichocarpa* associated with *A. thaliana*, *Salix purpurea*, and *O. sativa* was constructed based on the syntenic orthologous gene pairs. According to MCScanX analysis, six syntenic orthologous gene pairs were identified between *P. trichocarpa* and *A. thaliana*, seven gene pairs between *P. trichocarpa* and *O. sativa*, and 23 gene pairs between *P. trichocarpa* and *S. purpurea*, implying that *P. trichocarpa* has different directions in evolution with *A. thaliana*, *O. sativa* and *S. purpurea* (Figure 5). Those results indicated that the poplar and willow had a closer relationship [37]. The inter-and intra-genomic collinearity analysis suggested that segmental duplication or whole-genome duplication of orthologous gene pairs occupied a significant proportion in the evolutionary process of the ANN gene family.

### 2.7. Interaction Prediction and GO Enrichment Analysis

**Figure 6:**
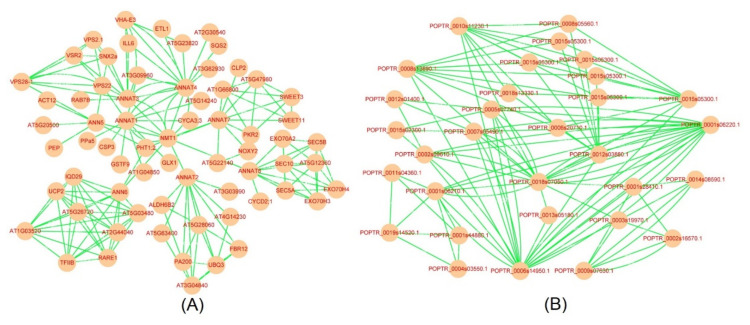
The putative proteins interacted with ANNs. The String database and Cytoscape were used to identify the interaction relationship of ANN members in Arabidopsis (**A**) and poplar (**B**).

Interaction prediction can reveal the putative relationship among proteins. In general, the interacting proteins may play an essential role in plant growth and development and response to various stresses by comprehensive regulation. The String database (https://string-db.org/ (accessed on 22 August 2021)) was used to identify the possible interaction network, and the Cytoscape software was applied to visualize. As shown in Figure 6A, the interaction network of AtANN members was relatively complicated, and the putative proteins that interacted with AtANNs contained ubiquitin-associated (UBA)/TS-N domain-containing protein, cold shock domain protein, glutathione S-transferase, glycylpeptide N-tetradecanoyltransferase, calcineurin-like metallo-phosphoesterase, and so on. Additionally, the interaction relationship of PtANNs indicated that PtANNs might interact with glutathione S-transferase, glycylpeptide N-tetradecanoyltransferase, and so on (Figure 6B). The putative interaction relationship revealed that ANNs might regulate cellular ROS and the balance of intracellular and extracellular permeability. 

**Figure 7:**
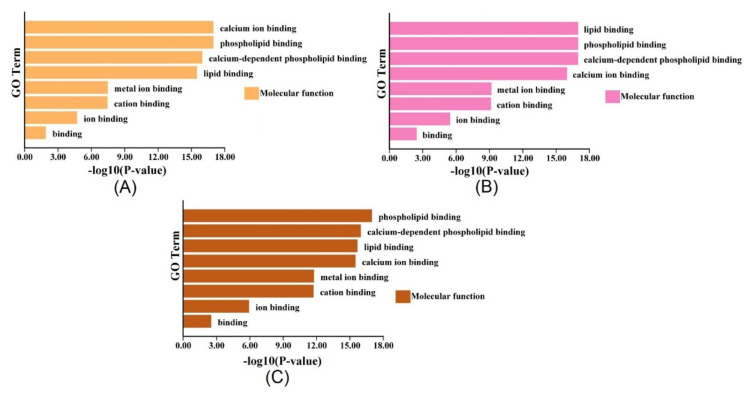
Gene ontology (GO) analysis of *ANN* genes from Arabidopsis (**A**), rice (**B**), and poplar (**C**) and distribution in categories of molecular function.

Regarding ANNs associated with ROS and osmotic homeostasis, gene ontology (GO) was applied to identify the putative physiological function of ANNs. Based on the GO analysis, the ANNs were significantly enriched in molecular function, such as calcium ion binding function, phospholipid-binding function, calcium-dependent phospholipid binding function, and lipid-binding function (Figure 7). It is suspected that ANNs may participate in osmotic stresses by regulating osmolytes, including lipid and ion. In addition, ANNs as the Ca^2+^-dependent proteins were supposed to be associated with the stress resistances through maintaining intracellular and extracellular Ca*^2+^* homeostasis. These observations have shown that ANNs are essential proteins for cells to resist intracellular and extracellular homeostasis. 

### 2.8. Cloning and Subcellular Localization of PtANNs

Gene-specific primers for *PtANN* CDS applications were designed based on the *PtANN* sequences (Supplementary Table S4). Using *P. trichocarpa* cDNA as a template, the target *PtANN* genes were amplified using PCR (Supplementary Figure S5). Additionally, the PCR products of *PtANN* genes were purified, and each *PtANN* gene fragment was ligated with PEASY-T3 plasmid, respectively. Then, the positive clones containing *PtANN* genes were screened and sequenced (Supplementary Figure S6).

**Figure 8:**
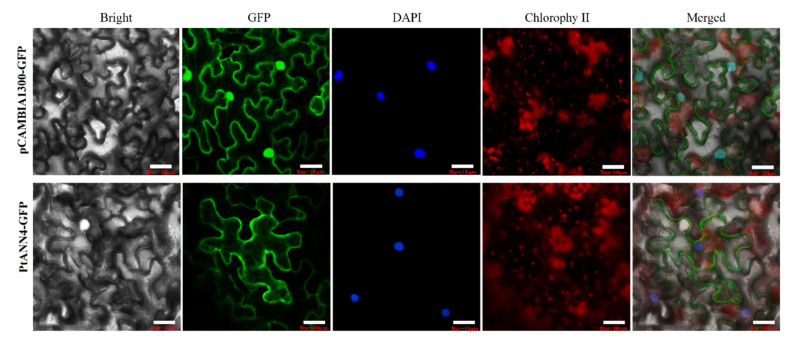
Subcellular localization of PtANN4. Transient expression of pCAMBIA1302-PtANN4-GFP vector in tobacco leaf cells with the pCAMBIA1302-GFP construct used as the control. Bar = 10 μm.

To further discover the function of PtANNs, the PtANN4 homologous with AtANN1 was chosen to examine the subcellular localization. The *PtANN4* was cloned into the pCAMBIA1300-GFP plasmid to generate the recombinant plasmid pCAMBIA1300-PtANN4-GFP. Subsequently, the recombinant plasmid transformed into Agrobacterium strain GV3101 was used to infiltrate tobacco leaves. The subcellular localization of PtANN4-GFP was identified 72 h after infiltration using a C2-ER confocal laser fluorescence microscope (Nikon, Tokyo, Japan). Subcellular localization showed that GFP signals (control group) were detected in the whole cells, while the fluorescence of the GFP emitted by PtANN4-GFP fusion protein was localized in the cytoplasm and plasma membrane of tobacco cells (Figure 8). Taken together, the result suggested that PtANN4 is a cytoplasm and plasma membrane-colocalized protein.

### 2.9. Tissue-Specific Expression Profile of *PtANNs*

**Figure 9:**
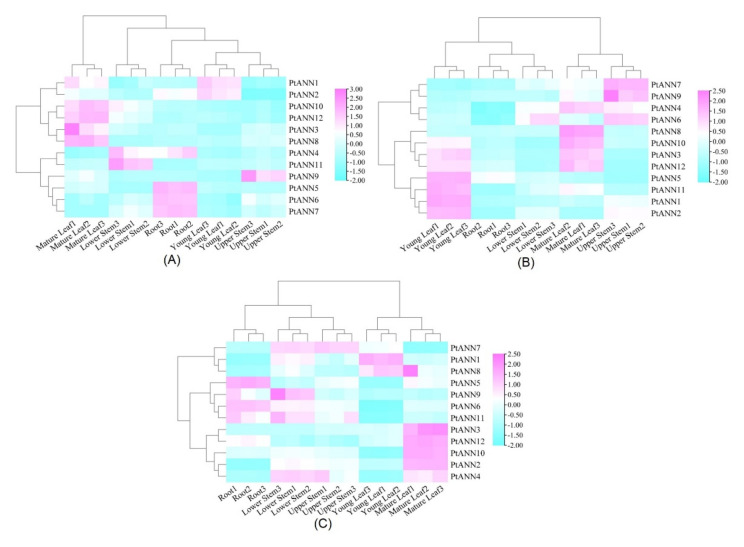
Tissue-specific expression patterns of *PtANN* genes in *P. trichocarpa* (**A**), ‘Nanlin 895’ (**B**), and ‘Shanxinyang’ (**C**) tissues. Three independent experiments were repeated, and poplar *PtActin* (XM-006370951) was used as an internal control.

To explore the putative functions of PtANNs, qRT-PCR was used to examine the expression patterns of *PtANN* genes in different tissues of *P. trichocarpa*, ‘Nanlin 895’, and ‘Shanxinyang’. Generally speaking, the heatmap in Figure 9 showed that the 12 *PtANN* genes were expressed in the roots, stems, and leaves of poplar, and *PtANN* genes are presented at the dominant tissue-specific expression. (Figure 9A). Additionally, the highest expression levels *PtANN4*, *9*, and *11* were identified in stems of *P. trichocarpa*. In contrast, the largest expression accumulations of *PtANN5–7* were found in roots, and the highest mRNA accumulations of *PtANN2* were detected in young leaves of *P. trichocarpa*. In addition, *PtANN1*, *3*, *8*, *10*, and *12* were highly expressed in mature leaves of *P. trichocarpa* (Figure 9A). Cluster analysis showed that *PtANN10* and *PtANN12* were clustered on the same branch. In contrast, *PtANN1–9* and *PtANN11* were clustered on another branch, implying that *PtANNs* clustered on the same branch had relatively similar expression patterns. The expression patterns of the *PtANN* genes were also discovered in the leaves, stems, and roots of ‘Nanlin 895’ (Figure 9B). For example, *PtANN6*, *7*, and *9* had relatively higher expression levels in stems. In comparison, other *PtANNs* presented higher expression levels in leaves, and the lower expression levels of all *PtANNs* were detected in roots. For ‘Shanxinyang’, the *PtANNs* expression shared the divergent features in different organs and tissues (Figure 9C). For example, higher transcriptional levels of *PtANN5* and *6* were presented at roots. *PtANN1–3*, *8*, *10*, and *12* exhibited higher expression levels in mature leaves. In addition, higher expression levels of *PtANN4*, *7*, *9*, and *11* were accumulated in the stems. Taken together, *PtANNs* are widely expressed in various tissues, indicating that PtANNs may be extensively involved in several types of physiological activities of poplar. In addition, the expression pattern of the same *PtANN* gene in different poplar varieties occupied the divergent feature, indicating that the same PtANN may participate in various physiological processes and performs other functions in different poplar varieties.

### 2.10. Expression Analysis of *PtANNs* under Abiotic Stress

**Figure 10:**
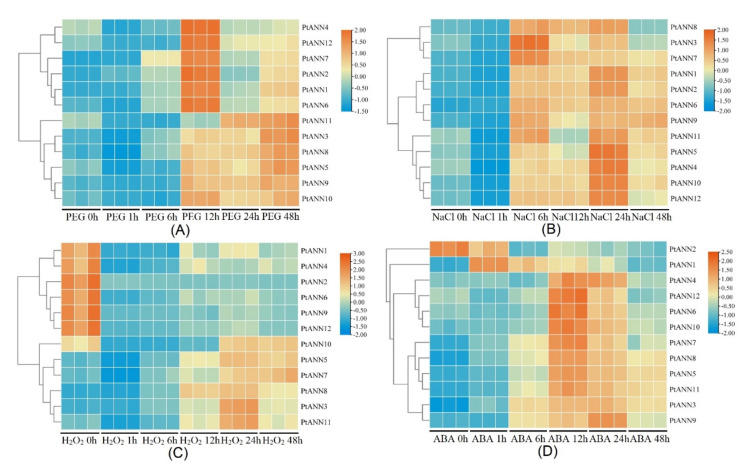
The qRT-PCR identification of *PtANN* expression levels in response to various stresses. The heatmap was used to show *PtANN* gene transcript levels under 10% PEG_6000_ (**A**), 200 mM NaCl (**B**), 2 mM H_2_O_2_ (**C**), and 200 μM ABA (**D**). Three independent experiments were repeated, and poplar *PtActin* (XM-006370951) was used as an internal control. *PtANN* expression was normalized to that in the untreated leaf.

To sufficiently investigate PtANNs function in abiotic stress, the 12 *PtANNs* expressions were analyzed by qRT-PCR experiments. The leaves of ‘Nanlin 895’ were treated for salt, drought, ABA, and PEG treatments. The expression of all *PtANNs* was up-regulated under 10% PEG_6000_ stress for most of the treatment time, and the transcript levels of *PtANN1*–*3* and *6*–*8* were distinctly accumulated when poplars were treated by PEG_6000_ stress. All those observations illustrated that PtANNs might be involved in the response of PEG_6000_ stress (Figure 10A). Moreover, the expression level of *PtANNs* was increased with 200 mM NaCl treatment. The most significant expression of *PtANN1*, *2*, and *7* were detected in 200 mM NaCl treatment (Figure 10B). In addition, the mRNA accumulations of *PtANN1*, *2*, *4*, *6*, *9*, and *12* were down-regulated under the 2 mM H_2_O_2_ treatment. While The expression levels of *PtANN3*, *5*, *7*, *8*, and *11* increased significantly after 2 mM H_2_O_2_ stress treatment. These results indicated that PtANNs have differentiated in response to H_2_O_2_ stress. The detailed regulation mechanism of PtANNs in response to ROS needs to be further explored (Figure 10C). Moreover, except for *PtANN2*, the transcript levels of *PtANNs* experienced large numbers of accumulation when the poplars were treated by 200 μM ABA stress. The highly significant expression accumulations were identified in *PtANN3*, *5*, *7*, *8*, and *11* under the most stressful time. Therefore, most PtANNs could respond to ABA treatment, implying that PtANNs may regulate stress response through the ABA signaling transduction pathway (Figure 10D). The above observations indicate that PtANNs that are considered the key regulators are possibly involved in various stresses.

## 3. Discussion

Abiotic stresses usually affect cell homeostasis and even result in cell death, which seriously affects plant development and output [38]. Plants have a series of regulatory mechanisms to withstand extremely adverse environments and maintain relative stability of the internal cell environment during evolution [39,40]. ANN plays an essential role in maintaining Ca^2+^ homeostasis, especially the stability of Ca^2+^ in the plasma membrane [13]. Plant ANN is a large and conserved gene family, which plays a vital role in plant growth and development and participates in various stress responses such as cold, drought, and salt resistance [26,41]. At present, the studies on the reaction of plant ANN to abiotic stress have partially been analyzed. To date, the research mainly focuses on herb plants such as Arabidopsis, rice, and cotton. However, the evolutionary history and characterization of ANN remain unknown in woody plants. In the present study, the poplar ANN gene family was identified based on the poplar genome, and annotation and the transcript patterns of *PtANNs* were also analyzed, providing a theoretical basis for studying the physiological mechanism and PtANNs function.

The cluster analysis found that the evolutionary relationship of plant ANN is complex, and ANNs from different species are distributed at intervals, with PtANNs interspersed among them. PtANNs are clustered with Arabidopsis (dicotyledon) ANNs, and a small part of PtANNs are pressed with rice (monocotyledon), implying that the evolutionary complexity of the PtANN gene family. Jami et al. [42] constructed a phylogenetic tree of 149 plant ANNs and divided them into nine groups. The observation implied that the expansion of the ANN gene family might be associated with genome complexity. Additionally, the phylogenetic tree indicated that the ANN gene family might be related to gene duplication, and *ANN* expansion means that the ANN family may play an essential role in response to environmental stress [43]. *P. trichocarpa* is a diploid plant with clear genetic information and contains 12 *PtANN* genes in the whole genome, more than *ANN* genes in *A. thaliana*. It is speculated that poplar has a relatively larger genome than *A. thaliana*. The *ANN* gene replication may occur with the evolution of species and the complexity of the plant genome, which increases the number of ANN family members in a species. The ANNs from monocotyledons and dicotyledons are clustered in different evolutionary subbranches, which indicates that their common ancestor produced species specificity in the process of evolution. Poplar ANNs have a close relationship with Arabidopsis ANNs, but a small part of poplar ANNs and rice ANNs are clustered together. The number of *PtANN* exons is mostly six, and few members occupy the four or five exons. It is speculated that this phenomenon results from combining two exons into one exon. At the same time, many members of *PtANNs* are distributed on the same chromosome, and their sequences and protein structures have high similarities.

For analysis of the PtANN conservative domain, it was found that PtANN4–7 contains the IRI motif and GXGT motif. Previous studies showed that IRI could promote the combination of plant ANN and F-actin. F-actin participates in many essential physiology activities in plant growth and development, such as cell secretion, cell division, cell morphology maintenance, and material transportation [10,44]. In relation to the ANN domain-containing IRI motif, it was speculated that this kind of ANN participant in plant regulatory processes through F-actin. Three of the eight members of the Arabidopsis ANN family (AtANN3, AtANN4, and AtANN4) do not contain IRI motifs [10], indicating that not all ANN members have IRI motifs, and this type of ANN may participate in plant physiological activities in other regulatory ways. PtANN4–7 contains a conserved IRI domain, implying that PtANN4–7 involved in plant physiological regulation are closely associated with domain IRI and F-actin. At the same time, PtANN1–3 and 8–12 may apply other regulatory mechanisms during the whole poplar lifestyle. In addition, the analysis of AtANN1 showed that the conserved His40 resembled the structure of horseradish peroxidase plays an essential role in Arabidopsis peroxidase activity [10,45]. The previous study on the crystal structure of cotton GhAnn1 indicated that the GhAnn1 MCCY domain named as S3 cluster is formed by two Cys, Met, and Tyr, which can be used as one of the receptors of the electron transport chain to participate in intracellular redox reaction [46]. At present, many studies have proved that ANN has peroxidase activity in plant cells. The recombinant *C. annuum* AnnCa24 and mustard AnnBj1 had peroxidase activity [20,21,47], and maize ANN showed peroxidase activity under pH 7.4 without calcium ion [48].

Moreover, maize, cotton, and tomato ANNs have proved that ANNs have the function of binding nucleotides and are involved in nucleotide hydrolyzation [21,49]. ANNs of maize and tomato can hydrolyze ATP and GTP, and the reaction rate is similar, but their affinity for GTP is significantly lower than that of cotton AnnGh1. The Walker A (GXXXXGKT/S) and GTP binding domain (GXXG), considered as a typical structure of GTPase, plays a vital function in ANN nucleotide-binding and hydrolyzation [49]. The cotton AnxGh2 and maize AnxZm33/35 showed that the GTP-binding motif partly overlaps with the Ca^2+^-binding site on the repeat IV, indicating that Ca^2+^ and GTP have a competitive relationship and Ca^2+^ can inhibit the GTPase activity of annexin [49,50]. In this study, the PtANN1, 3–5, 7, and 9 were identified to occupy the GXXG domain, indicating that PtANNs might have a similar function to cotton AnxGh2 and maize AnxZm33/35. However, the exact role of PtANN in nucleotide binding and hydrolyzation needs to be furtherly explored. 

Subcellular localization of a protein is usually associated with its function [51,52]. ANN has been considered a cytoplasmic protein with two soluble and stable forms. ANNs reversibly bound to cytoskeletal components or mediated the protein interaction between cells and extracellular matrix. Plant ANNs were identified as localized in the cytoplasm, plasma membrane, and nuclear membrane. Additionally, the more significant proportion of plant ANNs was illustrated as localized in the cytoplasm, and the possible reason was that ANNs play an essential role in membrane function [53,54]. In addition, the subcellular localization of plant ANNs was closely associated with the concentration of Ca^2+^ and pH in the cytoplasm [55]. In Arabidopsis, AnnAt2, AnnAt3, and AnnAt6 were located in the cytoplasm and nucleus, and AnnAt5 were located in peroxisome [10]. The wheat ANNs, P39, and p22.5 were localized to the plasma membrane under low-temperature stress [24]. Alfalfa MtAnn1 was located in the nuclear membrane [56]. Celery ANN, named VCaB42, was localized in the vacuolar membrane [57]. In this study, all PtANNs were predicted as located in the cytoplasm, and PtANN4 homologous with AtANN1 was identified as distinctly localized in the plasma membrane. A part of the GFP signal was detected in the cytoplasm. The plasma membrane was considered an essential interface between plant intracellular and extracellular environments, regulating various development and metabolic pathways. PtANN4 as a dominant plasma membrane binding protein may be well adapted to sense and respond to environmental stimuli.

The gene expression patterns are dominantly related to gene function, and there were significant differences in the expression patterns of *ANNs* in divergent plant tissues. The previous studies showed that the plant *ANNs* expression has a tissue-specific pattern [10,36,58]. Arabidopsis *AtANN1* was expressed in all tissues and highly accumulated in stems. Still, the *AtANN2* expression level in stems was relatively lower than that in other tissues, and a higher transcript level was identified in roots *AtANN5*, *AtANN6*, and *AtANN7* were expressed preferentially in flowers [10]. The expression of plant *ANN* is related to plant growth and development, involving embryogenesis, seed germination, root development, vascular bundle development, cork formation, cotton fiber elongation, and cell cycle [10,59,60]. Wheat *TaAnn10* gene was specifically expressed in anthers, related to wheat male fertility [61]. The difference in the *ANN* expression pattern reflects the difference in the functional division of annexin members. In this study, 12 *PtANN* genes were identified in different tissues of *P. trichocarpa*, ‘Nanlin 895’, and ‘Shanxinyang’, revealing that *PtANNs* are ubiquitously expressed in various tissues of poplar. In addition, the expression of *PtANN4*, *9*, and *11* dominantly accumulated in stems of *P. trichocarpa*. The relatively higher expression levels of *PtANN6*, *7*, and *9* were presented in stems of ‘Nanlin 895’, and the higher mRNA levels of *PtANN4*, *7*, *9*, and *11* were illustrated in stems of ‘Shanxinyang’. The same *PtANN* gene in different poplar varieties showed divergent expression patterns, implying that PtANNs undergo functional differentiation in the process of evolution. ABA and H_2_O_2_, as the critical stress signal in plant cells, are widely involved in plant growth and development or abiotic stress [62]. They are composed of systemic signal transduction and respond to abiotic stress through complex gene regulation networks [63,64]. The previous studies showed that environmental factors, such as drought, salt, lower temperature, and plant hormone, have distinct influences on the plant *ANNs* expression [36,65]. The Arabidopsis and rice *ANN* genes expression and abundance were regulated by drought stress [12,23,36]. For example, the Arabidopsis *AtANN1* and *AtANN4* expression levels were associated with the salt stress response [36]. The transcript level of rice *OsANN3* was also induced by PEG and ABA treatment [22]. Additionally, the expression levels of 8 tomato *SlANNs* were influenced by plant hormones, both *SlANN3*, *6*, *8*, and *9* were affected by ABA treatment, and the mRNA accumulations of *SlANN 1.1*, *1.2*, *4*, and *7* were induced by GA treatment [17]. In addition, six peanut *AhANNs* might respond to drought, salt, low-temperature, and hormones treatments [25]. In this study, we distinguished the expression levels of 12 *PtANNs* that could be affected by diverse abiotic stresses, which suggested that PtANNs might possess abiotic stress resistance based on the analysis of qRT-PCR. Additionally, the divergence within expression levels and abundances under the diverse abiotic stresses implied that PtANNs occupied different regulation modes in resistance to various treatments. Drought and salt stress can accumulate peroxide in plants, damage plant cells, and even cause many tissues and cell death. ANNs have peroxidase activity that plays an essential role in maintaining the stability of membrane structure and cell secretion. The objective was to explore the putative features and functions of PtANNs and provide a basis for improving the quality of poplars by mining the dominant PtANN resources.

## 4. Materials and Methods

### 4.1. Genome-Wide Identification of *PtANN* Genes

The whole genomes and annotations of *P. trichocarpa*, *A. thaliana*, and *O. sativa* were applied to downloaded from the phytochrome (https://phytozome-next.jgi.doe.gov/ (accessed on 2 August 2021)). The HMM model PF00191 of PtANN domain and 8 AtANN sequences were downloaded from TAIR (The Arabidopsis Information Resource) as a query to search the *P. trichocarpa* genome protein databases. The PtANNs achieved from the above methods were considered candidates and determined the completeness of the PtANN domain with SMART and the CDD database. In addition, the Cell-PLoc 2.0 (http://www.csbio.sjtu.edu.cn/bioinf/Cell-PLoc-2/ (accessed on 2 August 2021)) was used to identify the putative subcellular localization of PtANNs. The ExPASy (https://web.expasy.org/protparam/ (accessed on 2 August 2021)) was applied to calculate the molecular weights (MWs), theoretical isoelectric points (pI), and grand averages of hydropathicity (GRAVYs) of PtANNs.

### 4.2. Phylogenetic Tree and Three-Dimensional (3D) Structures of PtANNs

The software ClustalX2 was applied to perform the multiply alignment of PtANNs. The MEGA7 was used to construct phylogenetic trees using the neighbor-joining (NJ) method. The evolutionary relationships of the phylogenetic tree were calculated by performing the bootstrap values, tree inferred from 1000 replicates at each branch. The SWISS-MODEL (https://swissmodel.expasy.org (accessed on 2 August 2021)) was committed to constructing the homologous structures of PtANNs, and Chimera software was applied to visualize the 3D structures of PtANNs. The MEME program was used to identify motif structures of PtANN proteins, and the TBtools were used to visualize the conserved motifs and *PtANN* gene structures based on the poplar genome and innovation.

### 4.3. Promoter Cis-Acting Elements and Collinearity Analysis of *PtANNs*

The 2 kb upstream region sequences of *PtANNs* were extracted and submitted to the PlantCARE (http://bioinformatics.psb.ugent.be/webtools/plantcare/html/ (accessed on 2 August 2021)), and the TBtools was used to visualize *cis*-acting elements in *PtANN* promoters. The chromosomal distribution information of *PtANN* genes were obtained from *P. trichocarpa* genome database and genome gff3 file. Both *PtANN* gene duplication events, including tandem and segmental duplication and collinearity among different species, were analyzed by MCScanX and visualized by TBtools [66], and the values of Ka (nonsynonymous), Ks (synonymous), and Ka/Ks were calculated by TBtools/Simple Ka/Ks calculator.

### 4.4. Plant Materials and Various Abiotic Treatments

Poplar strains including *P. trichocarpa*, ‘Shanxinyang’ (*P. davidiana*× *P. bolleana* Loucne), and ‘Nanlin 895’ (*P. deltoides*× *P. euramericana*) were cultivated in a greenhouse under long-day conditions at 23 °C and 74% humidity. The *P. trichocarpa*, ‘Shanxinyang’, and ‘Nanlin 895’ were applied to investigate the transcript levels of *PtANNs* in different tissues. Additionally, ‘Nanlin 895’ was chosen to identify the expression patterns of *PtANNs* in response to various abiotic stresses. The poplar seedlings were treated individually with 200 mM NaCl, 10% PEG_6000_, 2 mM H_2_O_2_, and 200 μM ABA, and the poplar leaves were sampled at certain treatment times and stored at −80 °C until following RNA extraction.

### 4.5. *PtANN* Amplifications

Poplar RNA was extracted from the leaf before and after various stress treatments, stem, and root samples by RNA extraction kit (Takara, Tokyo, Japan) based on the instruction. The first-strand cDNA was synthesized using the 1 µg total RNA as template and reverse-transcriptase (Takara, Tokyo, Japan). To clone a full-length CDS of *PtANNs*, the gene-specific primers (Supplementary Table S4) were designed based on the poplar genome annotation. The PCR reaction procedure was followed: pre-denaturation at 95 °C for 5 min; followed by 35 cycles of denaturation at 95 °C for 1 min, annealing at 56 °C for 30 s, and extension at 72 °C for 30 s; and a final extension at 72 °C for 10 min. Subsequently, PCR products were ligated into the PEASY-T3 vector (TransGen, Beijing, China) and sequenced. 

### 4.6. Subcellular Localization of PtANN4

Based on the analysis of restriction sites of *PtANN4* and pCAMBIA1302, the *Kpn*I and *Xba*I were chosen to construct the subcellular plasmid. The gene-specific primers for constructing the recombinant vectors are shown in Supplementary Table S4. The coding region of *PtANN4* was cloned into the pCAMBIA1302 vector to generate the recombinant vector pCAMBIA1302-PtANN4-GFP. The Agrobacterium GV3101 containing the recombinant vector pCAMBIA1302-PtANN4-GFP were cultivated, harvested, and resuspended in 10 mM MgCl_2_ solution containing 10 mM 4-morpholineethanesulfonic acid hydrate (MES). The resuspended Agrobacterium GV3101 suspensions were infiltrated into *N. benthamiana* leaves, and the GFP signals were detected by a C2-ER confocal laser fluorescence microscope (Nikon, Tokyo, Japan) after injection for 72 h.

### 4.7. Identification of *PtANN* Expression Levels

The reverse-transcribed cDNA template was mixed in the total 20 µL reaction volume by UltraSYBR Green I Mixture (CWBIO, Beijing, China). The qRT-PCR analysis was performed on ABI 7500 Fast Real-Time PCR System (Applied Biosystems). The primers designed for the *PtANN* expression analysis were listed in Table S4. The Ptactin (XM-006370951) as an internal reference was used to identify the relative expression levels of PtANN genes through 2–ΔΔCT method. The amplification conditions were as follows: 95 °C for 10 min; followed by 40 cycles of 95 °C for 10 s, 60 °C for 30 s, and 72 °C for 30 s.

## 5. Conclusions

In the present study, the 12 *PtANNs* were systemically identified from *P. trichocarpa* genome, and gene application, gene and protein structures, chromosomal localization, and expression patterns were comprehensively illustrated. The 12 PtANNs could be divided into three major classes based on the phylogenetic tree. The PtANN structures and motif distributions shared high similarities with ANN in the same phylogenetic clade. Additionally, *PtANNs* presented tissue-specific expression patterns. In addition, PtANNs might play an essential role in the resistance of various abiotic stresses. These observations provide a theoretical basis and valuable information for further exploring the regulation mechanism of PtANNs in drought and salt stresses.

## Supplementary Materials

The following are available online at https://www.mdpi.com/article/10.3390/ijms23010515/s1.

## Abbreviations

annexin	ANN
abscisic acid	ABA
neighbor-joining	NJ
protein kinase C	PKC
three-dimensional	3D
actin-binding domain	IRI
gibberellin	GA
salicylic acid	SA
methyl jasmonate	MeJA
Pyrabactin resistance 1	PYR1
PYR1-like	PYL
protein phosphatase 2C	PP2C
sucrose non-fermenting1-related protein kinase2	SnRK2
protein kinase C	PKC
mitogen-activated protein kinase	MAPK
cyclic AMP	cAMP
glutathione S-transferase	GmGST
high temperature and high humidity	HTH
cyclin-dependent kinase 5	Cdk5
protein kinase A	PKA
cyclin-dependent kinase 5	Cdk5
glycogen synthase kinase 3	GSK3
DNA-dependent protein kinase	DNA-PK
ribosomal S6 kinase	RSK
division cycle2	cdc2
casein kinase II	CKII
