# Supplementary figures and images for: Genome-Wide Characterization and Abiotic Stresses Expression Analysis of Annexin Family Genes in Poplar
Source: Int J Mol Sci. 2022 Jan 3;23(1):515. doi: 10.3390/ijms23010515 (PMC8745089; doi:10.3390/ijms23010515)

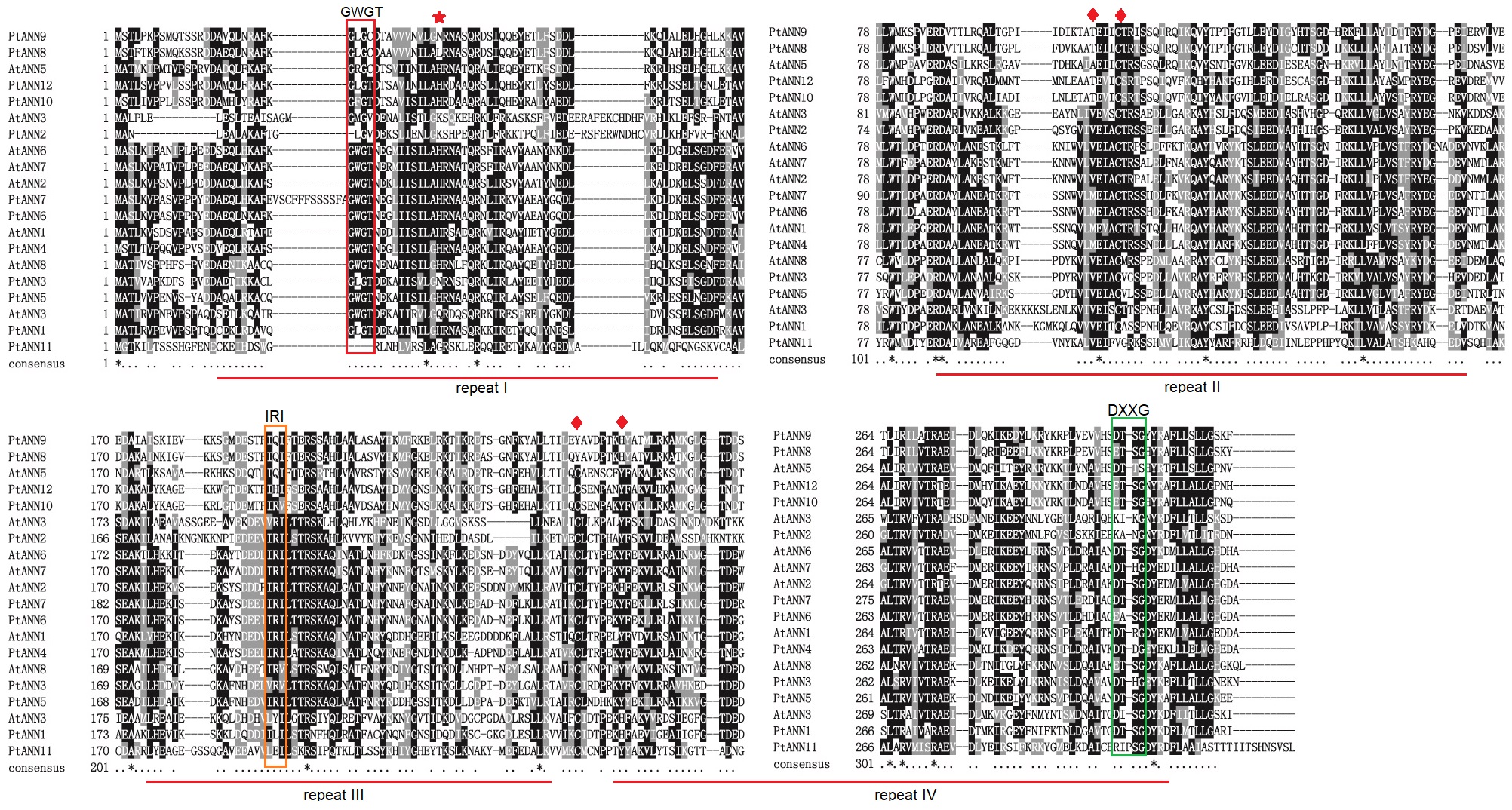

Supplement: Supplementary file 1 [file ijms-23-00515-s001.zip › Supplemental Figure S1.jpg]

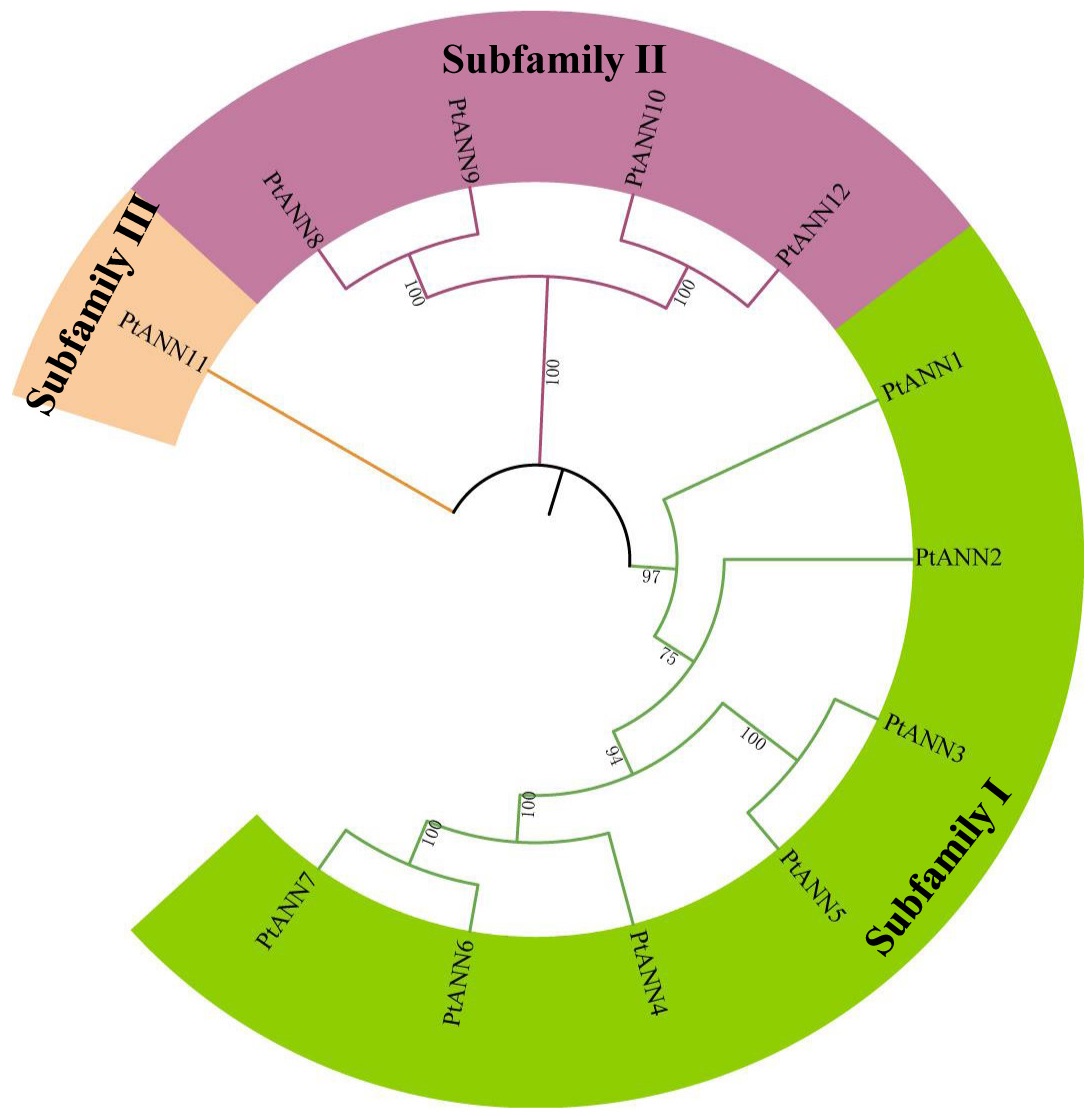

Supplement: Supplementary file 1 [file ijms-23-00515-s001.zip › Supplemental Figure S2.jpg]

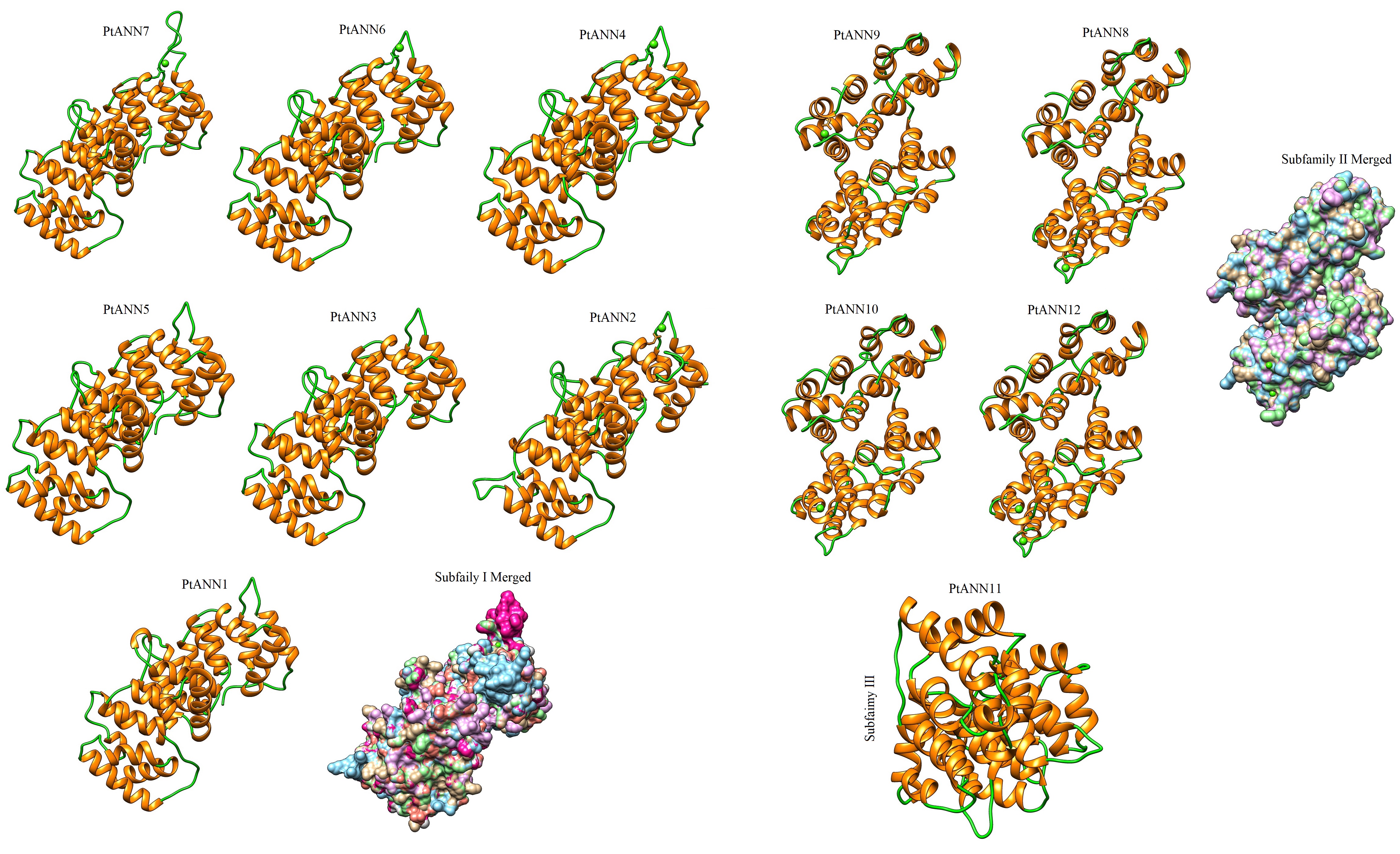

Supplement: Supplementary file 1 [file ijms-23-00515-s001.zip › Supplemental Figure S3.jpg]

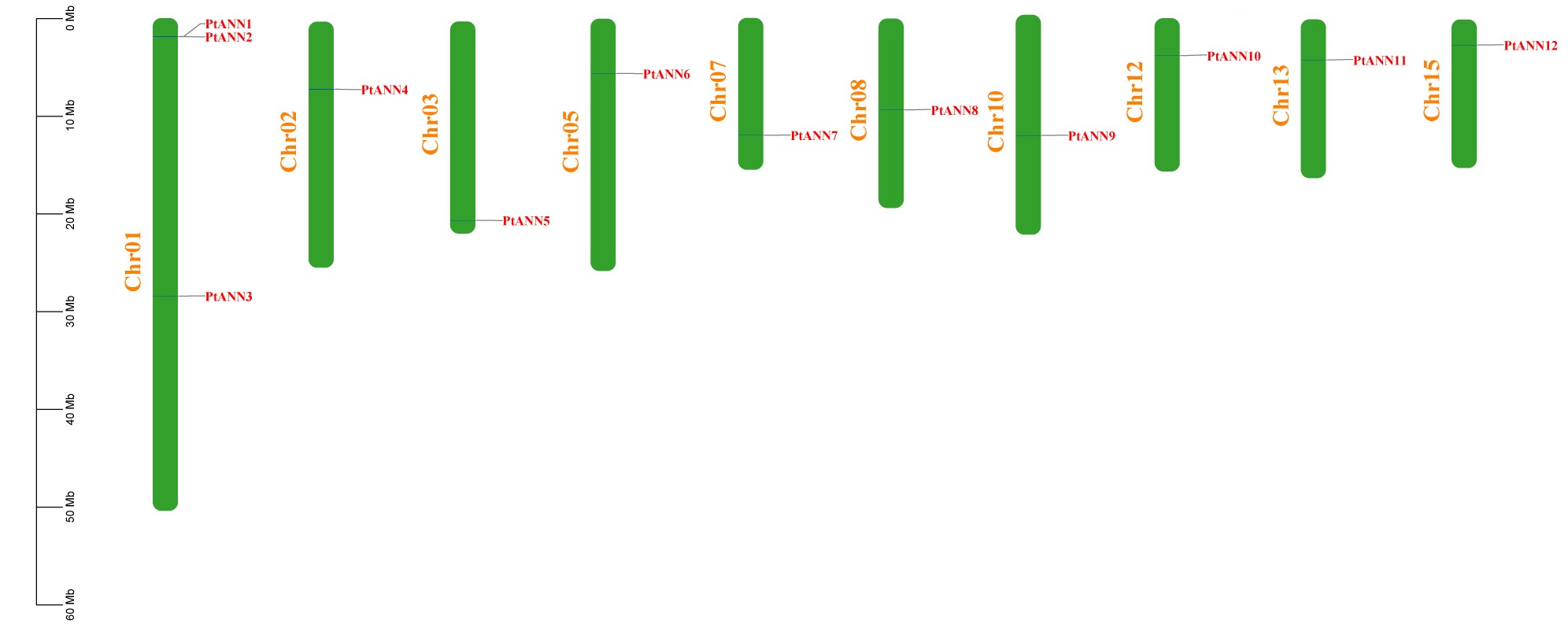

Supplement: Supplementary file 1 [file ijms-23-00515-s001.zip › Supplemental Figure S4.jpg]

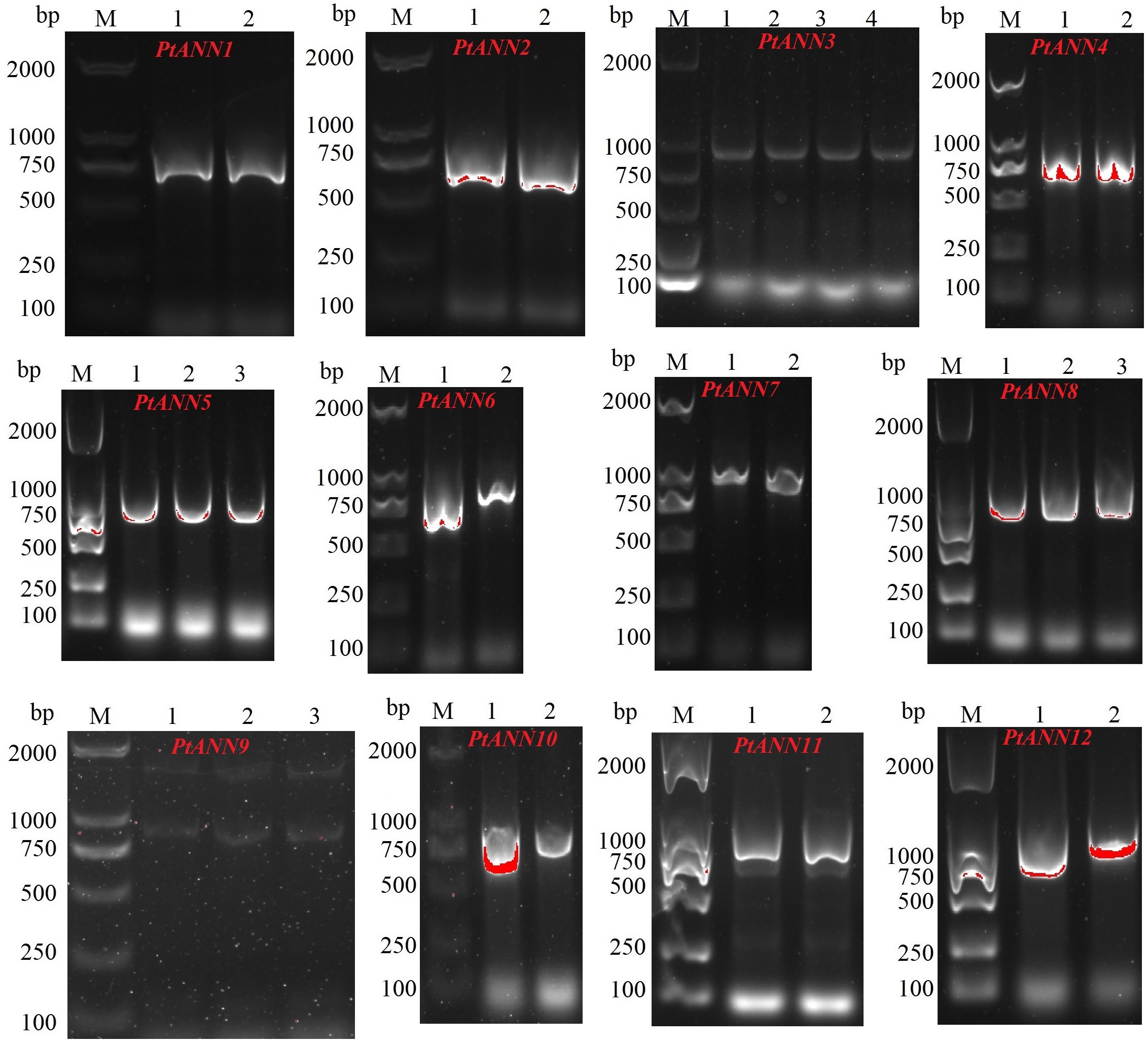

Supplement: Supplementary file 1 [file ijms-23-00515-s001.zip › Supplemental Figure S5.jpg]

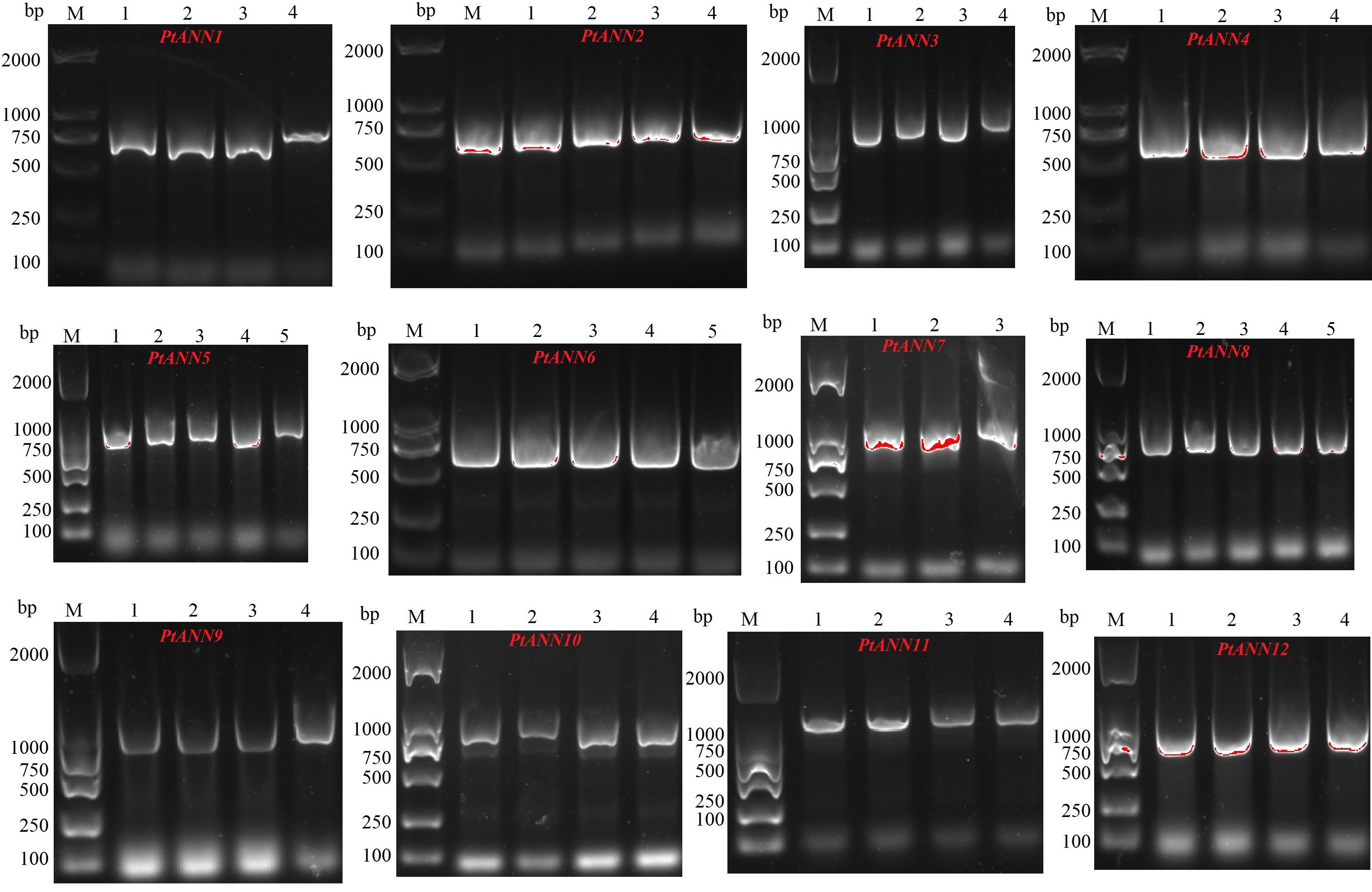

Supplement: Supplementary file 1 [file ijms-23-00515-s001.zip › Supplemental Figure S6.jpg]
